# Dogslife: A cohort study of Labrador Retrievers in the UK

**DOI:** 10.1016/j.prevetmed.2015.06.020

**Published:** 2015-12-01

**Authors:** C.A. Pugh, B.M.de C. Bronsvoort, I.G. Handel, K.M. Summers, D.N. Clements

**Affiliations:** The Roslin Institute and Royal (Dick) School of Veterinary Studies, University of Edinburgh, Easter Bush, Midlothian, Scotland, EH25 9RG, UK

**Keywords:** Dog, Cohort, Labrador Retriever, Morphology, Lifestyle, Exercise

## Abstract

Studies of animals that visit primary and secondary veterinary centres dominate companion animal epidemiology. Dogslife is a research initiative that collects data directly from owners about the health and lifestyle of Kennel Club (KC) registered Labrador Retrievers (LR) in the UK. The ultimate aim is to seek associations between canine lifestyle and health. A selection of data from Dogslife regarding the height, weight and lifestyle of 4307 LR up to four years of age is reported here.

The majority of the dogs were household pets, living with at least one other pet, in families or households with more than one adult. The dogs typically ate diets of dried food and daily meal frequency decreased as the dogs aged. Working dogs spent more time exercising than pets, and dogs in Wales and Scotland were exercised more than their counterparts in England. Dogs in households with children spent less time exercising than dogs in other types of households. There was considerable variation in height and weight measurements indicative of a highly heterogeneous population. The average male height at the shoulders was 2–3 cm taller than the UK breed standard. Dog weights continued to increase between one and four years of age. Those with chocolate coloured coats were heavier than their yellow and black counterparts. Greater dog weight was also associated with dogs whose owners reported restricting their dog’s exercise due to where they lived.

These findings highlight the utility of wide public engagement in the collation of phenotypic measures, providing a unique insight into the physical development and lifestyle of a cohort of LRs. In combination with concurrently collected data on the health of the cohort, phenotypic data from the Dogslife Project will contribute to understanding the relationship between dog lifestyle and health.

## 1 Introduction

In human medicine, it has been well demonstrated that lifestyle has health impacts, such as links between smoking tobacco and lung cancer (Doll and Hill, 1950), or exercise levels and mortality (Irwin et al., 2011). Understanding how people live and seeking associations between their lifestyle and health can facilitate investigations of disease mechanisms, which in turn may suggest avenues for intervention. Medical professionals are able to give patients evidence-based guidance on how to best maintain their health. By contrast in academic literature regarding canine health, there is a paucity of the most basic lifestyle information; knowledge about what is ‘normal’ for a dog in the UK is missing. Collecting lifestyle information and linking lifestyle with health is an obvious avenue for future exploration.

The disease burden of dogs visiting veterinarians in the UK is currently being assessed by two large-scale projects, SAVSNET (SAVSNET, 2014a) and VetCompass (VetCompass, 2014). Both have automated the collection of electronic records directly from veterinary practices and SAVSNET also collects diagnostic test results from laboratory facilities. SAVSNET quoted the number of individual pets involved in the project between September 2012 and February 2014 to be over 89,000 (SAVSNET, 2014b) and the running total on the VetCompass website in September 2014 (VetCompass, 2014) indicated that they had information relating to the veterinary care of over 800,000 dogs. Both of these projects have great scope to investigate disease in dogs seen at veterinary practices. However, they cannot gather information about illnesses that do not precipitate veterinary visitation and do not address the environment dogs are kept in, nor other relevant data such as diet and exercise regimes.

There is not just a lack of information regarding how dogs live, but also about the dogs themselves. The morphology expected of pedigree dogs is set out in the breed standards (The Kennel Club, 2014a). Standards such as these have been used to show that smaller breeds have greater longevity (Li et al., 1996, Adams et al., 2010) but exhibit more behaviours that might be considered undesirable (McGreevy et al., 2013). However, it is not known how many pedigree dogs actually meet the specified breed standard. If the breed standard is an ideal rather than a reality, then a major input of such analyses would not represent individual subjects, reducing the chances of finding associations.

A more detailed understanding of dog lifestyle and morphology would facilitate future studies. Initial results regarding a cohort of LR will be reported here with the aim of initiating investigations of the impact of lifestyle and morphology on dog health and well being.

## 2 Materials and methods

The study was approved by the Veterinary Ethical Review Committee of the University of Edinburgh.

A detailed description of the recruitment process is available in Clements et al. (2013). To summarise, puppies were initially registered with the KC by the breeder, and buyers of these puppies could transfer the registration after purchase. Breeders and new owners who transferred the registration of eligible dogs (born since 1st January 2010) received an A5 flyer about Dogslife with their registration information from the KC. There were two nightly electronic file transfers from the KC to Dogslife: firstly a list of all newly registered dogs (their KC identifier, sex, coat colour and date of birth) and secondly the names of all new owners who transferred their dog’s registration (for example ‘Miss A. Smith’). If the owner gave permission for their contact details to be shared, the second file transfer included the owners’ email and/or postal address. These owners were then emailed and sent postcards by Dogslife, as permitted, encouraging them to register via the project website (www.dogslife.ac.uk). Registration included giving basic information about the household, and a questionnaire (©The University of Edinburgh) was subsequently used to gather information on dog height, weight, exercise levels, diet and health. Data collected up to and including 31st December 2013 were used to describe the growth, health and lifestyles of LR up to the age of four years in the UK.

### 2.1 Questionnaire detail

Participants were prompted to complete the online questionnaire every month for the first year of their dogs’ lives and quarterly thereafter. Individual questions are detailed in Appendix A. All questionnaire answers or ‘data entries’ were automatically date-stamped. With the exception of dog weight, all questions required an answer before the owner could continue through the questionnaire. However, if the owner chose ‘other’ from a drop-down list, a free-text box would be generated and this could be left blank.

Measurements taken by owners included the height of their dog to the shoulder until the dog was 18 months of age (demonstrated via an online video). They were also asked to weigh their dog when possible, irrespective of age. Owners were asked to weigh their dogs’ meals then report the average daily food intake in addition to meal frequency and type of diet (for example ‘dried’ or ‘home-prepared’). Use of SI units in the UK is inconsistent so owners were given the option to enter a measurement and choose their preferred units from a drop down box (centimeters (cm) or inches for height, kilograms (kg) or pounds for dog weight and grams (g) or ounces (oz) for food weight). Entries made in inches were automatically multiplied by 2.54 and stored in cm. Entries made in pounds were divided by 2.20 and stored in kg. Entries made in ounces were multiplied by 28.3 and stored in g.

The data collected in the first 22 months of the project were validated through a series of owner visits and sampling of veterinary records (Pugh et al., 2015).

### 2.2 Statistical analyses

Data were extracted from the Dogslife database using the *RMySQL* package (James and DebRoy, 2012) and analyses were undertaken using R 3.0.2 (R Core Team, 2013). Linear mixed models were built using the *nlme* package in R (Pinheiro et al., 2013). Autocorrelation structures were used and owner and dog identities included as random terms to account for repeated measures. Reported models had the lowest Akaike Information Criterion (AIC) of all possible models, found using the *MuMIN* package (Bartoń, 2014). Assumptions of normality and homogeneity were checked by visual inspections of plots of residuals against fitted values.

### 2.3 Owner profiles

Associations were sought between different household characteristics. Multiple Chi-squared tests were undertaken assessing, for example, whether household type ‘retired’ and household types ‘not retired’ or household type ‘family’ and household types ‘not family’ were associated with different types of pet ownership (tests performed for all household types). Conservative Bonferroni corrections were applied to account for multiple testing.

Household location details were captured as postcodes and compared with available postcodes of eligible owners. Postcode area recruitment rates were determined and plotted using *maptools* in R (Bivand and Lewin-Koh, 2015). Postcode areas comprise the first letter(s) from the postcode, for example, EH25 9RG and G20 0SP would be in areas EH and G respectively.

### 2.4 Owner retention

Return intervals were examined and time to assumed loss from the project was investigated with a Cox proportional hazards model (Cox, 1972), using the *survival* package in R (Therneau, 2014). For dogs under one year of age, this was considered to be two months after their last questionnaire answer and for dogs over one year, four months. After model fitting, the proportional hazards assumption was tested. The percentages of dogs aged over one, two and three years that were retained within the project were reported.

### 2.5 Exercise

A weighted average of weekday (5/7) and weekend day (2/7) exercise levels was created. Total daily times spent exercising (TDE) were generated by taking the midpoints of the relevant exercise time categories (the ‘over 2 h’ category was assumed to be ‘2–4 h’) and summing. These times were square-root transformed (tTDE) before further analysis. Univariable plots were created comparing tTDE in different groups. A multivariable, linear mixed model was built considering associations between tTDE and age, season, dog purpose, household type, location and concurrently reported exercise restrictions. Age was considered as both a continuous and categorical predictor. Seasons were defined as groups of three consecutive months with Winter comprising December, January and February. In addition to the main effects model, biologically plausible interactions between age and other factors were considered in a more complex model.

### 2.6 Dog heights

**Fig. 1:**
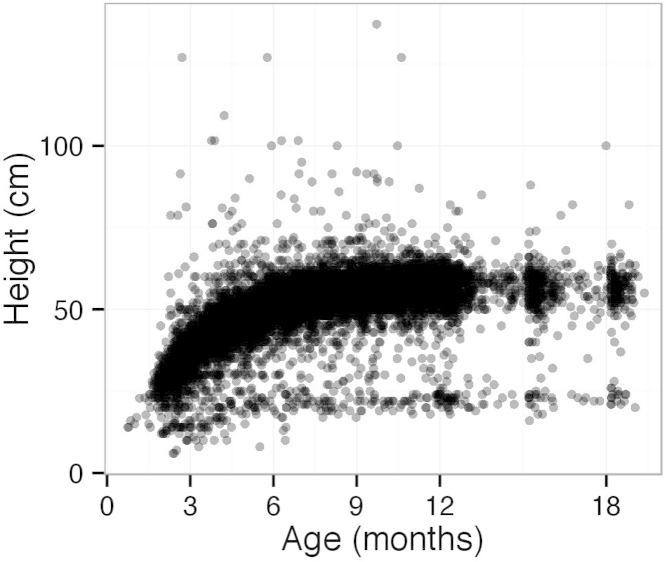
Raw heights of all dogs plotted against their ages.

Early explorations were undertaken of the raw, database-recorded heights of the cohort as they aged (Fig. 1). There were two distinct growth curves and it was hypothesised that the lower curve, which was approximately 2.5 times shorter than the main curve, was generated by owners who had taken measurements in inches but reported them as cm. It was also thought possible that some of the very high heights were measured in cm and reported in inches.

A probabilistic model was used to estimate whether entries might have been made in the correct or incorrect units. Eqs. (1)–(3) describe the heights which were assumed to be normally distributed with a mean height that changed exponentially with age. Each height would also fit one of three classes: measured in cm and reported in inches, measured and reported in the same units, measured in inches and reported in cm.

(1) height=N(μ,τ)

(2) $$\mu_{i}=a\{1-e^{\left(-b({\text{age}}_{i}c)\right)}\}\times {\text{class}}_{i}$$

(3) $$\text{class}=\left(\begin{array}{c} \frac{1}{2.54}\\ \begin{array}{c} 1\\ 2.54 \end{array} \end{array}\right)$$

The model required Bayesian priors, shown in Eqs. (4)–(9). Parameter *a* is the mean full height of the dogs and was taken from the UK KC breed standard for LR which was 55–56 cm for females and 56–57 cm for males (The Kennel Club, 2014a). Parameter *b* is a proxy for growth rate. The height was growing half way closer towards its maximum height, *a*, every ln2/*b* days. Parameter *c* is an offset term that allowed the height to have a non-zero value when the pups were born. Parameter *pi* is the prior probability of a measurement belonging to each different error class: i.e. estimated 10% chance of being subject to each type of inches-cm error and 80% chance of having the correct units. Once identified, the mis-reported heights were corrected using a multiplier of 2.54 or 1/2.54.

(4) a=N(56,0.01)

(5) b=Uniform(0,1.5)

(6) c=Uniform(0,100)

(7) τ=Gamma(0.001,0.001)

(8) $$sd=\sqrt{\frac{1}{\tau }}$$

(9) pi=Dirichlet(0.1,0.8,0.1)

The model was estimated under a Bayesian framework using the *rjags* package (Plummer and Stukalov, 2014). Each sex was modelled separately. One thousand iterations were used for adaptation and 2000 were discarded as ‘burn-in’. The final model was based on a further 5000 iterations and the mixing of the models was checked to ensure that sufficient iterations had been performed using the *coda* package (Plummer et al., 2006).

### 2.7 Dog weights

Weights of dogs over one year were explored using a linear mixed model. The focus of the model was on main effects but biologically plausible interactions between age, sex, neuter status and height were also assessed.

## 3 Results

### 3.1 Owner profiles

Between 1st January 2010 and 31st December 2013, 151,182 dogs were eligible to join Dogslife and names were passed to Dogslife for 83,532 owners who transferred their dog’s registration. Contact details were included for 50% (41,476/83,532) by email and 60% (50,109/83,532) by post; 62% (52,181/83,532) by at least one method. Assuming, in the absence of exact data, that each registered dog was associated with a single owner, contact details were available for the owners of just 35% of all eligible dogs.

The registered cohort comprised 4148 owners (7.9% of 52,181 contactable owners). Of those with titles that had clear gender definitions, 76.7% were female compared to just 53.6% of the 83,532 KC owners for whom names were available. Over 96% of Dogslife owners registered just one dog with the project; 127 owners had two dogs and a further 12 owners had registered three or more. Owners reported that the majority of their households comprised either families (45%; 1862/4148) or more than one adult (40%; 1673/4148) but there were also retired households (6.6%; 273/4148), single adults (5.3%; 218/4148) and some owners did not describe their household (2.9%; 122/4148). Owners from retired households were disproportionately more likely to give the project permission to contact them by telephone (*χ*^2^ = 20.96 (1df), *P* < 0.001).

**Fig. 2:**
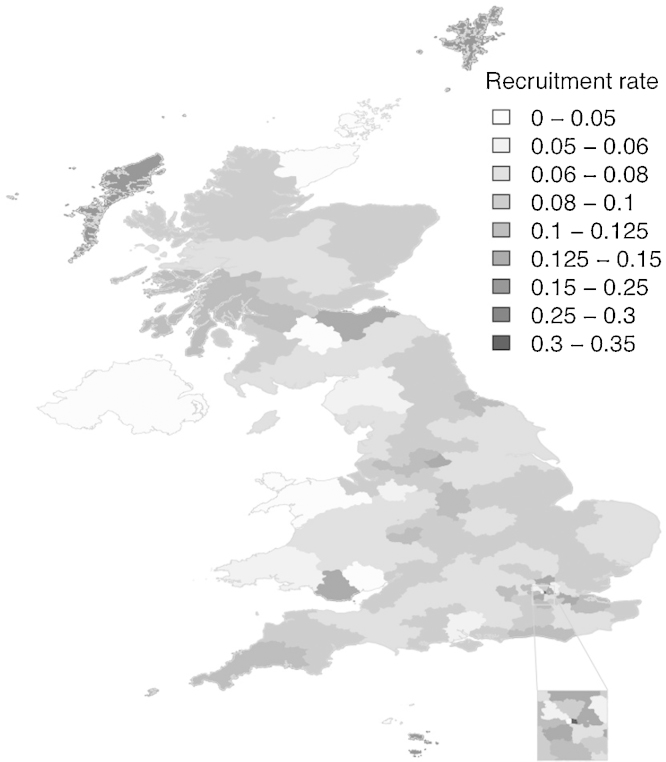
Map of Dogslife recruitment rates by postcode area. The denominator is not all eligble owners but rather, all eligible owners for which postcode data were available so the rates are over-estimates.

Location details were captured as postcodes and they break down as follows: England (78%; 3227/4148), Scotland (14%; 591/4148), Wales (3.6%; 151/4148), Northern Ireland (NI) (1.5%; 63/4148), Isle of Mann (0.22%; 9/4148), Jersey (0.12%; 5/4148), Guernsey (0.024%; 1/4148) and postcode not reported (2.4%; 101/4148). Fig. 2 shows UK-wide recruitment rates by postcode area. The denominator is not all eligible owners but the 50,109 for whom address details were available so the rates are overestimates.

Eighteen point two percent of Dogslife households included somebody who smoked tobacco (95% CI: 17.0–19.5%). Tobacco smoking prevalence for all individuals in the UK in 2013 was 19.1% (95% CI: 18.3–20.1%) (Orchard and Office for National Statistics, 2014). Households that did not report keeping any other pets (41%; 1719/4148) were in the minority. A simplified description of other pets kept in participating households is shown in Table 1. Families were disproportionately less likely to have another dog (*χ*^2^ = 13.7 (1df), *P* < 0.001) and disproportionately more likely to have a cat (*χ*^2^ = 48.4 (1df), *P* < 0.001) compared to other households. By contrast, households comprising more than one adult were disproportionately like to have no other pets (*χ*^2^ = 22.4 (1df), *P* < 0.001).

The results of an investigation into factors associated with assumed loss to the project are shown in Table 2. It should be noted that return intervals were irregular and many owners assumed to be lost were instead late. The maximum return interval was nearly three years, considerably more than the one or three months requested. Permission to contact owners by telephone and email both significantly improved the likelihood of those owners remaining with the project. Irrespective of contact preferences, retired households and those with another dog were disproportionately more likely to stay with the project. By contrast, family households were more likely to be lost to the project. Dog purpose was excluded from the final model as with inclusion, the proportional hazards assumption was violated. However assistance dogs were routinely lost at one year. They were typically guide dogs, registered by their puppy walker. At one year the dogs would be returned to Guide Dogs for the Blind for further training and officially leave Dogslife. Country location was not associated with loss to the project.

### 3.2 Dog profiles

There were 4307 registered dogs comprising 2041 females and 2266 males. Their reported coat colours were black (49%; 2121/4307), yellow (27%; 1167/4307), chocolate (21%; 898/4307), fox red (2.2%; 96/4307), hailstone (0.023; 1/4307), other (0.35%; 15/4307) and not reported (0.21%; 9/4307). Their main purposes were reported to be pets (68%; 2941/4307), working dogs (5.8%; 253/4307), assistance dogs (0.77%; 33/4307), multi-purpose (0.46%; 20/4307), show dogs (0.23%; 10/4307), breeding dogs (0.046%; 2/4307), other (0.56%; 24/4307) and not reported (24%; 1024/4307). The different reported purposes were disproportionately split between different types of households (Table 3). Working dogs were found disproportionately in households comprising more than one adult when compared to other household types (*χ*^2^ = 14.6 (1df), *P* < 0.001).

**Fig. 3:**
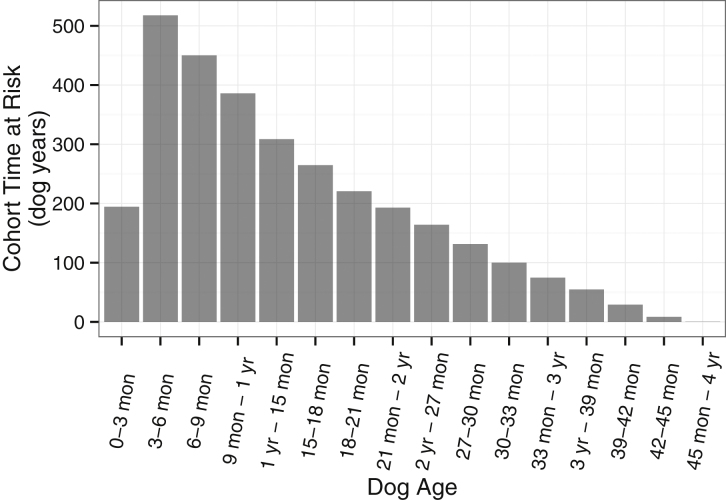
Cohort time at risk. A dog of precisely three months of age would lie in the 3–6 months category.

Completed questionnaires were available for 3249 of 4307 dogs, relating to a total of 3098 dog years at risk. After the loss of 1058 dogs between registration and initial questionnaire completion, there was ongoing loss to the project as the dogs aged. The percentages still up to date after the dogs reached one, two and three years old were 44% (1432/3255), 35% (722/2093) and 29% (235/822) respectively. These values increased to 60% (1432/2474), 43% (722/1692) and 36% (235/652) when the group of 1058 dogs were excluded. The median age of recruitment was 92 days and the time at risk is shown, split according to dog age, in Fig. 3.

### 3.3 Neutering

**Fig. 4:**
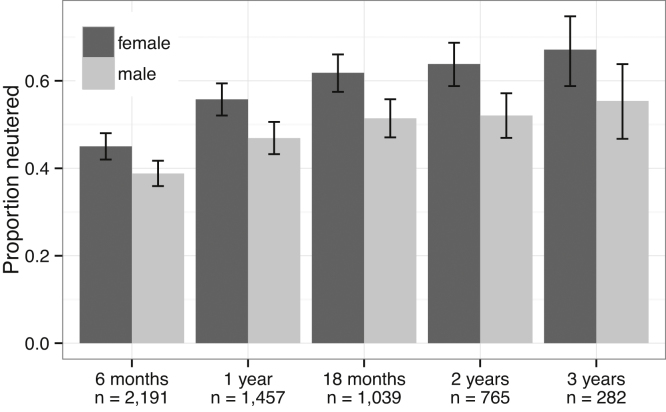
Cumulative neutering rates (with 95% CI) for cohort members that had associated data entries after each given age. For example, owners of 1039 dogs completed a questionnaire when their dog was aged over 18 months.

The neutering age distribution was right-skewed and the median ages were 282 days for males and 297 days for females (ranges = 35–1349 days and 33–1077 days respectively). Just 913 of 3249 dogs were reported to have been neutered giving a neutered population of 28.1% of the cohort. However, loss to follow-up appeared to be affecting the denominator value as only 2191 owners completed a questionnaire when their dog was aged six months or over. Fig. 4 shows the cumulative neutering rates for dogs whose owners answered the neutering question at different ages. The neutered proportion gradually increased with age because more were neutered and fewer were still in the project, contributing to the denominator.

### 3.4 Diet

**Fig. 5:**
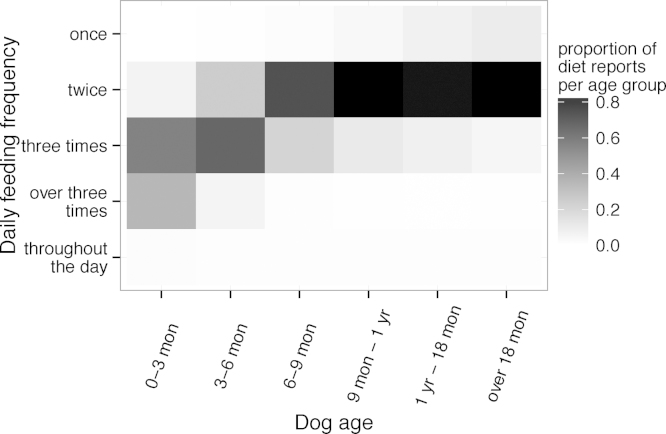
The proportion of dogs of each age group that ate at different frequencies daily. A dog of precisely three months of age would lie in the 3–6 months category.

Dietary data were collected for 3097 dogs, of which 2291 dogs had more than one report. The types of food were dried (80%; 12,124/15,219), a mixture of dried and wet (13%; 2005/15,219), raw (1.9%; 291/15,219), home prepared (1.1%; 171/15,219), wet (1.1%; 165/15,219) and other (3.0%; 463/15,219). The majority of dogs (1642 of 2291) did not have varying diet types; 1503 eating a consistent diet of dried food. The daily feeding frequency decreased as the dogs aged and settled at twice daily for most dogs at between six and nine months (Fig. 5).

### 3.5 Sleeping locations

Sleeping location data relating to 3251 dogs were divided as follows: indoors alone (55%; 9102/16,461), indoors with a person (and possibly another pet) (21%; 3499/16,461), indoors with another pet only (19%; 3156/16,461), and outside (possibly with another pet) (4.3%; 704/16,461). Of the dogs that had more than one questionnaire answered, 76.2% (95% CI: 74.0–78.3%) did not change their sleeping location.

Typically, dogs were not reported to sleep outside all of the time. There were yearly peaks in dogs sleeping outside in August 2011 and 2012 and July 2013. Dogs that slept outside at least once (5.1%; 166/3251) were disproportionately found in NI (Fisher’s exact test: odds ratio = 4.2, *P* = 4.9e-04) and much more likely to be working dogs (Fisher’s exact test: odds ratio = 163.23, *P* < 2.2e-16).

### 3.6 Exercise

**Fig. 6:**
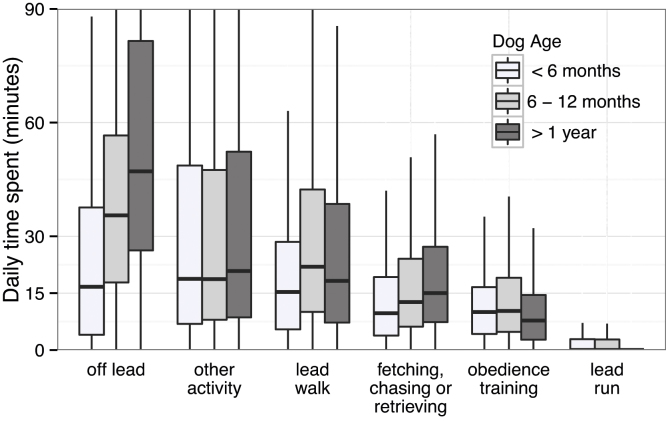
Boxplot of time spent exercising at different ages (cropped to show just the IQR).

Exercise data were collected for 3225 dogs, comprising 16,328 reports. The times spent on each exercise category were strongly right-skewed so Fig. 6 is cropped to show boxplots of the interquartile range (IQR) rather than the complete distribution. The majority of exercise time was spent ‘off lead’ and doing ‘other’ activities.

**Fig. 7:**
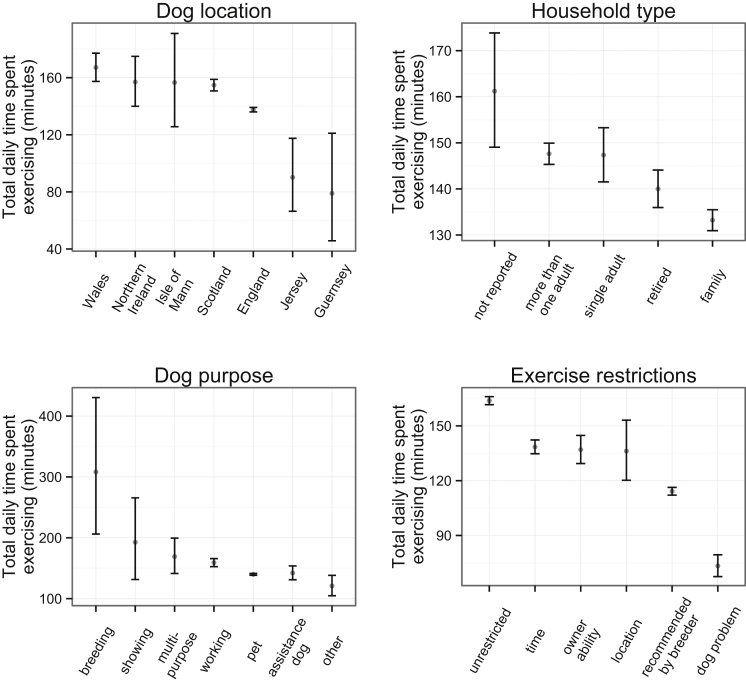
Variation in the daily time spent exercising. Group means with 95% confidence bars were generated from square root transformed data then re-squared for ease of interpretation.

The mean TDE was 157.5 min, the median was 128.7 min and the IQR was 84.4–200.9 min. In univariable analyses, country, dog purpose, exercise restrictions and household type were all associated with different amounts of tTDE (Fig. 7); time of year was not. However season was associated with tTDE in the multivariable model with the maximum amount of time spent exercising occurring in spring. The fixed effects of the multivariable model which excluded interaction terms are presented in Table 4. The random effect of ownership had an intercept standard deviation of 3.66 and the dog effect nested within the owner effect had an intercept standard deviation of 0.42. The correlation structure was autoregressive of order 1, with *ϕ* = 0.359. Age was not linearly related with exercise levels so the model included a categorical age measure. Dogs in families spent less time exercising than dogs in households with single adults or more than one adult and dogs in Wales and Scotland exercised more than those in England.

On examining models including interaction terms we identified a statistically significant effect that working dogs over six months of age spent more time exercising than household pets and the difference increased in dogs over one year. The results refer to tTDE and the increases were 0.49 and 0.68 min (approximately 4% and 6%), *P* = 0.03 and 0.009, for dogs aged between six months and one year and over one year respectively.

### 3.7 Dog heights

**Fig. 8:**
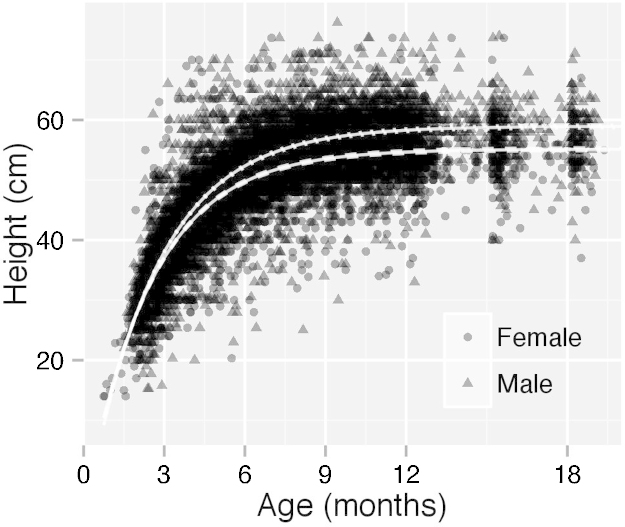
Dog heights corrected for assumed unit errors. Modelled growth curves are shown with 95% credible intervals for males (dotted) and females (dashed). The credible intervals are so close to the modelled growth curve that they appear to overlie them.

Extreme heights such as zero or one were excluded before modelling which resulted in the complete removal of some dogs. The model results, based on 3180 of 3249 dogs and 12,479 heights, are shown in Table 5. It was estimated that 470 heights had been reported in the wrong units. The maximum height for each sex (parameter a) would theoretically only be reached at an infinite age but the mean heights at 18 months were similar at 55.1 cm for females and 58.9 cm for males. The mean male height was 2–3 cm higher than the UK breed standard (The Kennel Club, 2014a) and there was wide variation in heights to the shoulder (*sd* = 4.67 and 5.01 cm for females and males respectively). Of all measurements of males over one year, only 12.9% (95% CI: 10.5–15.7%) met the breed standard. Even for females, whose average height fitted the UK standard, only 20.5% (95% CI: 17.6–23.6%) of measurements met the standard. The corrected data are shown in Fig. 8 with the modelled growth curves for males and females.

### 3.8 Dog weights

The dog weight model was based on 1049 dogs, 1016 owners and 4260 weights. The fixed effects parameters are shown in Table 6. None of the tested interaction terms improved the model. The random effect of ownership had an intercept standard deviation of 3.01 and the dog effect nested within the owner effect had an intercept standard deviation of 1.50. The correlation structure was autoregressive of order 1, with *ϕ* = 0.686, indicating a high degree of autocorrelation.

The total time spent exercising was not associated with dog weight but working dogs, a group that typically spent more time exercising than pets, were more than 2 kg lighter than pets. The mean weight of a two-year-old Dogslife LR was 26.8 kg for females and 31.6 kg for males. Both measurements fit within the suggested weight range for adults of the breed of 25–34 kg (Alderton and Morgan, 1993).

## 4 Discussion

Engaging thousands of dog owners in the Dogslife project has generated a wealth of data that begin to address knowledge gaps regarding UK LRs and their lifestyles. In order to generalise from the cohort, these data must be considered in the context of potential selection bias. Dogslife owners were disproportionately likely to be female. Males are often under-represented in surveys, for example Søgaard et al. (2004), so this imbalance is not atypical of a study whose participants were self-selecting. Reassuringly, Dogslife members were geographically distributed in proportion to LR KC registrations for whom address details were available and Dogslife household smoking rates were comparable to that reported for individuals in the UK. There was little evidence in terms of demographic factors that the recruited Dogslife cohort were unrepresentative of LR owners in the UK.

Retention bias was potentially more problematic as owners were being disproportionately lost to the project and dog age was correlated with many of the lifestyle factors. People who described their households as ‘families’ or whose household included a tobacco smoker were more likely to be lost to follow-up (Table 2). By contrast, retired households and those including another dog were more likely to be retained. Indeed, these two factors were themselves positively correlated within the cohort. In their examination of biases in a Spanish cohort study, Alonso et al. (2006) found a similarly increased risk of loss with regard to tobacco smokers and also that older people were more likely to be retained. With regard to the excess loss of families, it is possible that time constraints were a contributing factor because families were also a group who spent less total time exercising their dogs.

Of the data reported in this publication, the proportions neutered were likely to be the only measures that might be adversely affected by retention bias. For dogs whose owners ever answered the neutering question, just 28.1% of dogs were apparently neutered, but the denominator includes many dogs whose owners were effectively lost to the project before their dogs were old enough to be neutered. One would expect the prevalence of neutered dogs in the cohort to increase with age, as shown in Fig. 4, and the prevalence of neutering in Dogslife registered dogs over three years of age reached 0.67 for females and 0.55 for males. These values are considerably higher than 0.41 which was reported in recent work using the veterinary records of 148,741 dogs in the UK (O’Neill et al., 2014). This may reflect the differences between Dogslife’s population of KC registered pedigree dogs and the more mixed group examined by O’Neill et al. but may also indicate that owners who neuter their dogs were more likely to remain in the Dogslife study.

In terms of lifestyle factors, there was considerable homogeneity in the cohort. The majority ate dried food and slept alone. Individual dogs typically did not change diet type but the number of meals per day decreased as the dogs aged. The sleeping location reports highlighted a potential cultural difference between NI and the rest of the UK, with a higher proportion of dogs in NI sleeping outside at least once. NI had a similar mean temperature to both England and Wales in 2013 but had fewer hours of sunshine and more rain (Met Office, 2014) so this was unlikely to be associated with better climatic conditions. The association was found irrespective of dog purpose. From a human perspective, it was interesting that over 20% of reports involved the dog sleeping in the same room as a person. Sensitisation to inhaled dog allergens is one of the major risk factors for asthma (Custovic and Simpson, 2012) so this may have implications for the health of the owners.

Multiple factors were associated with the total daily time spent exercising. The exercise times of breeding, showing and multi-purpose dogs, and those located in Jersey, Guernsey and the Isle of Mann were based on too few dogs to draw sensible conclusions. Of the four largest regional contributors to the cohort, dogs in England spent less time exercising than dogs in Wales or Scotland. Unsurprisingly, working dogs spent more time exercising than pets and dogs whose owners reported that their exercise was restricted spent less time exercising than those whose exercise was unrestricted. The clearest difference was for dogs that had a problem, but owners that followed breeder recommendations also spent less time exercising their dogs. This latter type of exercise restriction was associated with younger dogs (unpublished results); younger dogs specifically spent less time ‘off lead’ and ‘fetching, chasing and retrieving’. It could be hypothesised that the young dogs were still learning to return to their owners when unrestricted or that breeders advised limiting exercise while the dogs were young because of perceived deleterious effects on musculoskeletal health. Such perceptions can be exemplified by advice from the Kennel Club (The Kennel Club, 2014b).

### 4.1 Dog weights

Nearly 30 years ago, LR were identified as the most likely breed to be overweight in the UK vet visiting dog population (Edney and Smith, 1986) and it is of concern that the average weight of the cohort continued to increase, approximately linearly, at 0.89 kg per year between one and four years of age. Whilst it is not possible to extrapolate beyond the age range of the data, if weight continues to increase markedly with age, an expanding proportion of the cohort will become subject to the health consequences of obesity. For example, it has been demonstrated in Elkhounds that there is an association between dogs that were overweight throughout their lives and diabetes mellitus (Wejdmark et al., 2011) and in LR, there is an association between higher body weight and increased prevalence and severity of hip dysplasia (Smith et al., 2006).

The weight model included some surprising results such as chocolate coloured LR being, on average, 1.39 kg heavier than their yellow and black counterparts and neutering apparently having minimal effect. A closer look at the weights associated with neutered and entire dogs indicated that only after the dogs reached three years of age did the weights of neutered dogs become greater than that of entire dogs and that there were not enough dogs of this age to affect the model parameters.

### 4.2 Dog heights

In 2008, Sutter et al. collected measurements for 1155 dogs including 14 LR and assessed the percentage of those measured that met the American KC (AKC) breed standards (American Kennel Club, 2014). It was concluded that the AKC breed standards were a good proxy for height at the shoulder. There is greater allowance for variation in the AKC standard for LR (5.08 cm for each sex in the USA compared to 1 cm for each sex in the UK) but there was also potential for bias in their study. The majority of their sample comprised dogs that had been entered in conformational competitions whereas few of the Dogslife cohort were show dogs. The issue of incorrect measurement or reporting must be considered with all Dogslife data (the height unit error being an obvious example) but visits to a sample of the cohort found no systematic bias to owner height measurements (unpublished results). Therefore whilst individual measurements might be treated with caution, the model parameters should be a good guide to the heights of the population.

Breed standard heights have been used as group phenotypes in studies as proxies for dog size. It is undoubtedly convenient and minimises the time and expense of data collection from individual dogs. However, the Dogslife results suggest two things: firstly that the breed standard does not necessarily reflect the average height for a breed and secondly, that even if it does represent the average, the variability of morphologies might mean that this average poorly reflects many individuals real morphologies. Under these circumstances, using the breed standard may not be appropriate and might limit the ability of investigators to find true effects. Studies, such as that by Frischknecht et al., (2013), that use individual dog measurements to characterise a phenotype, should have more scope to identify complex patterns. In that instance, it was possible to find potentially causative mutations associated with dwarfism in LR.

## 5 Conclusion

The morphological detail and lifestyle information collected by the Dogslife project offer a unique insight into the lives of pedigree LRs in the UK. These findings set a baseline for further analysis of the relationship between dog morphology, lifestyle and health. It is hoped that Dogslife will contribute to an evidence-based approach to healthy dog aging.

## Figures and Tables

**Table 1:** The relationship between pet ownership and household type for participants in the Dogslife project. Households that reported owning another dog, cat, other pet or did not report any pet (beyond their Dogslife registered dog), have been categorised by household type. Percentages are the percentage of each household type that reported having that type of pet. Individual households may appear up to three times in the table as they may, for example, own another dog, a cat and another pet.

	Another dog	(%)	Cat	(%)	Other^c^	(%)	Dogslife registered dog only	(%)
Family	521	(28.0^a^)	507	(27.2^b^)	430	(23.1^b^)	613	(32.9^a^)
More than one adult	564	(33.7^b^)	334	(20.0)	174	(10.4^a^)	767	(45.8^b^)
Retired	110	(40.3^b^)	41	(15.0)	9	(3.3^a^)	134	(49.1)
Single adult	84	(38.5)	36	(16.5)	24	(11.0)	92	(42.2)
Not reported	5	(4.1^a^)	4	(3.3^a^)	4	(3.3^a^)	112	(91.8^b^)
Total	1284	(30.9)	922	(22.2)	641	(15.4)	1718	(41.0)

a *χ*^2^ test performed with Bonferroni correction, negative association, *P* < 0.0025. For example, 28% (521 of 1862) of families reported having another dog compared with 33% (763 of 2286) for all other household types combined.

b *χ*^2^ test performed with Bonferroni correction,positive association, *P* < 0.0025. For example, 40% (110 of 273) of retired households reported having another dog compared with 30% (1174 of 3875) of all other household types combined.

c Other excludes dogs and cats but includes all other reported pets.

**Table 2:** Results of Cox proportional hazards model assessing loss to the project.

	Hazard ratio	95% CI		*P*-value
	*e^β^*	Lower	Upper	
Household types				
Family	1			
More than one adult	0.77	0.71	0.83	<0.001
Retired	0.47	0.4	0.56	<0.001
Single adult	0.81	0.69	0.95	0.01
Not reported	1.14	0.51	2.54	0.75

Smoking status				
Non-smokers	1			
Smokers	1.21	1.11	1.33	<0.001
Not reported	0.39	0.13	1.17	0.09

Postcode				
Full postcode	1			
First half only	0.68	0.17	2.62	0.57
Not reported	3.8	1.76	8.23	<0.001

Communications				
No telephone contact	1			
Telephone contact	0.55	0.51	0.59	<0.001
No email contact	1			
Email contact	0.44	0.39	0.51	<0.001
No newsletter subscription	1			
Newsletter subscription	1.3	1.18	1.44	<0.001

Other household pets				
No other dog	1			
Another dog	0.83	0.77	0.9	<0.001

**Table 3:** The numbers of each type of dog purpose reported by owners from different household types.

	Family	More than one adult	Retired	Single adult	Not reported
Household pet^a^	1288	1231	205	153	64^a^, ^c^
Working dog^a^	84^a^, ^c^	132^a^, ^d^	21	9	7
Assistance dog^b^	8	11	10^b^, ^d^	3	1
Multi-purpose^b^	7	9	2	2	0
Show dog	3	4	0	3	0
Breeding dog	1	1	0	0	0
Other^b^	8	8	2	4	2
Not reported^a^	515^a^, ^d^	350^a^, ^c^	47^a^, ^c^	61	51^a^, ^d^
Total	1914	1746	287	235	125

a *χ*^2^ tests performed with Bonferroni correction. For example, 84 of 1914 dogs in families were working dogs compared with 169 of 2393 in other household types. Due to low numbers in many categories, only household pet, working dog and purpose not reported categories were assessed for associations.

b Fisher’s exact tests performed with Bonferroni correction. For example, 8 of 1914 dogs in families were assistance dogs compared with 25 of 2393 in other household types. Due to very low numbers, show and breeding dog categories were not considered.

c Negative association, *P* < 0.003.

d Positive association, *P* < 0.003.

**Table 4:** Fixed parameters of model of square-root transformed total daily time spent exercising.

	Value	95% CI				*P*-value
		Lower		Upper		
Intercept	11.02	10.8		11.24		<0.001
Age category						
Under 6 months	0					
6 months – less than 1 year	1.36	1.24		1.48		<0.001
1 year and over	1.9	1.76		2.04		<0.001

Season						
Spring	0					
Summer	−0.1	−0.21		0.02		0.1
Autumn	−0.13	−0.25		−0.01		0.03
Winter	−0.18	−0.3		−0.07		1.50E-03

Dog purpose						
Household pet	0					
Working dogs	0.3	−0.15		0.7		0.21
Breed, show, multi-purpose dogs	0.61	−0.41		1.64		0.24
Assistance dogs	0.73	−0.39		1.85		0.2
Other purpose	−0.96	−2.33		0.42		0.17

Location						
England	0					
Wales	1.12	0.49		1.74		<0.001
Scotland	0.37	0.05		0.7		0.02
Northern Ireland	0.47	−0.49		1.42		0.34
Isle of Man	1.16	−1.12		3.44		0.32
Jersey	−0.68	−4.62		3.27		0.74
Guernsey	−2.18	−8.98		4.61		0.53
Location not reported	−0.02	−1.59		1.56		0.98

Household type						
Family	0					
More than one adult	0.47	0.22		0.72		<0.001
Single adult	0.72	0.19		1.25		7.60E-03
Retired	−0.21	−0.66		0.23		0.35
Household type not reported	1.09	−0.21		2.39		0.1

Exercise restrictions						
None	0					
Dog problem	−4.3	−4.56		−4.04		<0.001
Recommended by breeder	−1.08	−1.21		−0.95		<0.001
Owner ability	−0.83	−1.18		−0.48		<0.001
Time restrictions	−0.54	−0.72		−0.36		<0.001
Location	−0.6	−1.16		−0.03		0.04

**Table 5:** Height model parameters.

Variable	Female (95% CI)	Male (95% CI)
*a*	55.1 (54.9–55.4) cm	59.0 (58.7–59.2) cm
*b*	0.0132 (0.0128–0.0137)	0.0126 (0.0122–0.0131)
*c*	7.03 (4.43–9.63) days	9.37 (6.77–11.9) days
*sd*	4.67 (4.59–4.76) cm	5.01 (4.92–5.10) cm

**Table 6:** Fixed parameters of dog weight model (dogs of one year and over).

	Value	95% CI		*P*-value
		Lower	Upper	
Intercept	18.4	16.8	19.9	<0.001
Dog age (years)	0.89	0.76	1.02	<0.001
Height^2^ (cm)	2.20E-03	1.80E-03	2.70E-03	<0.001

Neuter status				
Entire	0			
Neutered	−0.12	−0.37	0.13	0.34

Coat colour^a^				
Black	0			
Chocolate	1.39	0.78	2	<0.001
Fox red	−0.84	−2.46	0.77	0.32
Yellow	0.19	−0.35	0.73	0.5

Dog sex				
Female	0			
Male	3.65	3.15	4.16	<0.001

Dog purpose				
Pet	0			
Working dog	−2.13	−3.01	−1.25	<0.001
Other^b^	2.49	0.75	4.24	9.60E-03

Owner smoking status				
Non-smoker	0			
Smoker	1.09	0.41	1.77	1.70E-03
Not reported	−1.4	−3.49	0.69	0.19

Other pets				
No other dog	0			
Another dog	−0.48	−0.99	0.03	0.07

Daily time spent exercising (h)				
Fetching, chasing and retrieving	−0.22	−0.35	−0.08	1.70E-03
Other	−0.09	−0.18	8.20E-03	0.07

Exercise restrictions				
None	0			
Owner location	0.95	0.33	1.57	2.80E-03
Owner ability	0.25	−0.13	0.63	0.2
Dog problem	−0.02	−0.34	0.3	0.89
As recommended by breeder	0.04	−0.18	0.25	0.74
Owner time	−0.19	−0.41	0.02	0.08
Daily food quantity (g)	5.70E-04	9.90E-05	1.10E-03	0.02

a The hailstone dog was treated as black and the KC registered colours were used for those that were unreported or reported as ‘other’.

b Other dog purpose included show, breeding, multi-purpose and all ‘other’ dogs. Assistance dogs were excluded because they typically left the project at one year.

## References

[bib0005] Adams V.J., Evans K.M., Sampson J., Wood J.L.N. (2010). Methods and mortality results of a health survey of purebred dogs in the UK. J. Small Anim. Pract..

[bib0010] Alderton D., Morgan T. (1993). Dogs.

[bib0015] Alonso A., Seguí-Gómez M., de Irala J., Sánchez-Villegas A., Beunza J.J., Martínez-Gonzalez M.A. (2006). Predictors of follow-up and assessment of selection bias from dropouts using inverse probability weighting in a cohort of university graduates. Eur. J. Epidemiol..

[bib0020] American Kennel Club, 2014. American Kennel Club Labrador Retriever Breed Standard [WWW Document]. URL https://www.akc.org/breeds/labrador_retriever/breed_standard.cfm (accessed 01.12.2014).

[bib0025] Bartoń, K., 2014. MuMIn: Multi-model inference version 1.10.5. http://cran.r-project.org/package=MuMIn.

[bib0030] Bivand, R., Lewin-Koh, N., 2015. maptools: Tools for Reading and Handling Spatial Objects version 0.8-34. http://cran.r-project.org/package=maptools.

[bib0035] Clements D.N., Handel I.G., Rose E., Querry D., Pugh C.A., Ollier W.E., Morgan K.L., Kennedy L.J., Sampson J., Summers K.M., de Bronsvoort B.M.C. (2013). Dogslife: a web-based longitudinal study of Labrador Retriever health in the UK. BMC Vet. Res..

[bib0040] Cox D. (1972). Regression models and life tables (with discussion). J. R. Stat. Soc. Ser. B.

[bib0045] Custovic A., Simpson A. (2012). The Role of inhalant allergens in allergic airways disease. J. Invest. Allergol. Clin. Immunol..

[bib0050] Doll R., Hill A. (1950). Smoking and carcinoma of the lung. Br. Med. J..

[bib0055] Edney A.T., Smith P.M. (1986). Study of obesity in dogs visiting veterinary practices in the United Kingdom. Vet. Rec..

[bib0060] Frischknecht M., Niehof-Oellers H., Jagannathan V., Owczarek-Lipska M., Drögemüller C., Dietschi E., Dolf G., Tellhelm B., Lang J., Tiira K., Lohi H., Leeb T. (2013). A COL11A2 mutation in Labrador retrievers with mild disproportionate dwarfism. PLoS One.

[bib0065] Irwin M.L., McTiernan A., Manson J.E., Thomson C.A., Sternfeld B., Stefanick M.L., Wactawski-Wende J., Craft L., Lane D., Martin L.W., Chlebowski R. (2011). Physical activity and survival in postmenopausal women with breast cancer: results from the women’s health initiative. Cancer Prev. Res..

[bib0070] James, D., DebRoy, S., 2012. RMySQL: R interface to the MySQL database version 0.9-3. http://cran.r-project.org/package=RMySQL.

[bib0075] Li Y., Deeb B., Pendergrass W., Wolf N. (1996). Cellular proliferative capacity and life span in small and large dogs. J. Gerontol. A Biol. Sci. Med. Sci..

[bib0080] McGreevy P.D., Georgevsky D., Carrasco J., Valenzuela M., Duffy D.L., Serpell J.A. (2013). Dog behavior co-varies with height, bodyweight and skull shape. PLoS One.

[bib0085] Met Office, 2014. Climate summary by region, 2013 [WWW Document]. URL http://www.metoffice.gov.uk/climate/uk/summaries/2013/annual/regional-values (accessed 10.12.2014).

[bib0090] O’Neill D.G., Church D.B., McGreevy P.D., Thomson P.C., Brodbelt D.C. (2014). Prevalence of disorders recorded in dogs attending primary-care veterinary practices in England. PLoS One 9, e90501..

[bib0095] Orchard, C., Office for National Statistics, 2014. Adult smoking statistics in Great Britain, 2013 [WWW Document]. URL http://www.ons.gov.uk/ons/dcp171778_386291.pdf (accessed 02.12.2014).

[bib0100] Pinheiro, J., Bates, D., DebRoy, S., Sarkar, D., Team, R.D.C., 2013. nlme: Linear and Nonlinear Mixed Effects Models version 3.1-111. http://cran.r-project.org/package=nlme.

[bib0105] Plummer, M., Stukalov, A., 2014. rjags: bayesian graphical models using MCMC version 3-13. http://mcmc-jags.sourceforge.net.

[bib0110] Plummer M., Best N., Cowles K., Vines K. (2006). CODA: convergence diagnosis and output analysis for MCMC. R News.

[bib0115] Pugh C.A., Summers K.M., Bronsvoort B.M.C., Handel I.G., Clements D.N. (2015). Validity of internet-based longitudinal study data: the elephant in the virtual room. J. Med. Internet Res..

[bib0120] R Core Team, 2013. R: A language and environment for statistical computing version 3.0.2. http://www.r-project.org/.

[bib0125] SAVSNET, 2014. SAVSNET [WWW Document]. URL http://www.savsnet.co.uk/ (accessed 15.09.2014).

[bib0130] SAVSNET, 2014. SAVSNET Demographics [WWW Document]. URL http://www.savsnet.co.uk/savsnet-reports/ (accessed 15.09.2014).

[bib0135] Smith G.K., Paster E.R., Powers M.Y., Lawler D.F., Biery D.N., Shofer F.S., McKelvie P.J., Kealy R.D. (2006). Lifelong diet restriction and radiographic evidence of osteoarthritis of the hip joint in dogs. J. Am. Vet. Med. Assoc..

[bib0140] Søgaard A.J., Selmer R., Bjertness E., Thelle D. (2004). The Oslo health study: the impact of self-selection in a large, population-based survey. Int. J. Equity Health.

[bib0145] Sutter N.B., Mosher D.S., Gray M.M., Ostrander E.A. (2008). Morphometrics within dog breeds are highly reproducible and dispute Rensch’s rule. Mamm. Genome.

[bib0150] The Kennel Club, 2014. Labrador Retriever Breed Standard [WWW Document]. URL http://www.thekennelclub.org.uk/services/public/breed/standard.aspx?id=2048 (accessed 26.06.2014).

[bib0155] The Kennel Club, 2014. Puppy and dog walking tips [WWW Document]. URL http://www.thekennelclub.org.uk/getting-a-dog-or-puppy/general-advice-about-caring-for-your-new-puppy-or-dog/puppy-and-dog-walking/ (accessed 09.01.2015).

[bib0160] Therneau, T.M., 2014. A Package for Survival Analysis in S version 2.37-7. http://cran.r-project.org/package=survival.

[bib0165] VetCompass, 2014. VetCompass [WWW Document]. URL http://www.rvc.ac.uk/VetCompass/Index.cfm (accessed 15.09. 2014).

[bib0170] Wejdmark A.-K., Bonnett B., Hedhammar A., Fall T. (2011). Lifestyle risk factors for progesterone-related diabetes mellitus in elkhounds - a case-control study. J. Small Anim. Pract..

