# Supplementary material for: Dogslife: A cohort study of Labrador Retrievers in the UK
Source: Prev Vet Med. 2015 Dec 1;122(4):426–35. doi: 10.1016/j.prevetmed.2015.06.020 (PMC4674016; doi:10.1016/j.prevetmed.2015.06.020)
Supplement: Supplementary file 1 [file mmc1.docx]

Appendix 1: Relevant sections of Dogslife registration process and questionnaire (© University of Edinburgh).

1. Register your dog

* Required information

Your dog’s name *
(the name you call your dog rather than their registered Kennel Club name)
[free-text box]

What colour is your dog? *
[dropdown list of options: Black, Yellow, Chocolate, Fox Red, Other. If ‘Other’ is selected, a free-text box appears to state colour]

What sex is your dog? *
[select Male/Female]

What is your dog's date of birth? *
[free-text box with calendar pop-up, and help button with image of Kennel Club certificate circling where to find it on the form]

Please confirm your dog's date of birth? *
[free-text box with calendar pop-up]

Is your dog a Labrador Retriever? *
[select Yes/No]

What is your dog's Kennel Club Registration Number (2 characters followed by eight digits) *
[free-text box, and help button with image of Kennel Club certificate circling where to find it on the form]

Confirm your dog's Kennel Club Number *
[free-text box]

Users need to click Save & Continue to proceed to next page

2.1 Your Registration Details

* Required information

E-mail Address*
[free-text box]

Confirm e-mail Address*
[free-text box]

Please tick this box if you would prefer us **not** to contact you about your pet by e-mail:
[box to tick]

Please tick this box if you would prefer **not** to receive the Dogslife newsletter on a monthly basis:
[box to tick – if ticked next question disappears]

Please indicate which format you would like to receive the newsletter in:
[select Plain text/HTML]

Password (between 6 and 11 characters)
[free-text box]

Confirm Password
[free-text box]

Users need to click Save & Continue to proceed to next page

2.2 Your Profile

* Required information

Title*
[dropdown list: Mr, Mrs, Miss, Ms, Dr, Professor, Other. If ‘Other’ is selected, a free-text box appears to state title]

First Name*
[free-text box]

Surname*
[free-text box]

Daytime Contact Telephone Number (including STD code)*
[free-text box]

Please tick this box if you do **not** want us to contact you about your pet by telephone:
[box to tick]

Add second contact email? (optional)
Second e-mail
[free-text box]

Confirm second e-mail
[free-text box]

Users need to click Save & Continue to proceed to next page

2.3 Your Household

* Required information

Post Code (must be valid UK Post Code)*
[free-text box]

How would you describe your household? *
[dropdown list: Single Adult, More than one Adult, Family (one or more adult and one or more children), Retired (Single or Couple), Other. If ‘Other’ is selected, a free-text box appears to enter details]

Does anybody in the household smoke?*
[select Yes/No]

Are there other pets in the household?*
[select Yes/No. If ‘Yes’ selected question below appears]

What species and how many?
[dropdown list: Dogs, Cats, Other, and dropdown list of number. If ‘Other’ is selected, a free-text box appears. Option to add more so boxes appear again]

Users need to click Save & Continue to proceed to next page which reads:

**Thank you!**

Thanks for registering [dog name] with Dogslife.

Questionnaire

1.1 Pet Profile

* Required information

Has [dog name] been neutered?* (**What does neutering mean?**)
[link/help reads: Neutering is the surgical removal of your dog’s reproductive organs. It is also termed “Spaying” if your dog is female, or “Castration” if he is male. [More information on neutering](http://www.pethealthinfo.org.uk/dogs/caring.html)]
[select Yes/No]

How tall is [dog name]?* (**How do I measure my dog’s height?**)
[link/help shows video and written instructions and pictures]
[2 dropdown lists, one for number (1-100), and one to select cms/inches]
[note this question is asked until 18 months of age and then again at 3 years of age]

How much does [dog name] weigh? (**How do I measure my dog’s weight?**) [note not a required field]
[link/help to wording and pictures]
[free-text box to enter number, and dropdown list to select kg/lbs]

Do you own [dog name] primarily as a:*
[dropdown list: Household pet, Working dog (e.g. Gundog), Assistance Dog (e.g. Guide Dog), Other. If ‘Other’ selected, free-text box appears to enter details. This question should only be asked the first time the user completes the questionnaire, so should not appear the next times they complete the questionnaire]

Users need to click Save & Continue to proceed to next page

1.2 Pet Profile

* Required information

Where does [dog name] sleep at night?*
[dropdown list: Alone in a room in house, In a room shared with a person, In a room shared with a pet, In a room shared with a pet and a person, Outside, Other. If ‘Other’ is selected, a free-text box appears to enter the sleeping location]
[After 10 month data entry, this question should be pre-populated with previous answer]

Users need to click Save & Continue to proceed to next page

1.3 Pet Profile

* Required information

On average, in the last week, for how long does [dog name] do the following exercise(s) EACH DAY?

Walking on the lead:

Weekday? [dropdown list] Weekend day? [dropdown list]

Running on the lead:

Weekday? [dropdown list] Weekend day? [dropdown list]

Walking/running off the lead:

Weekday? [dropdown list] Weekend day? [dropdown list]

Exercise involving fetching, chasing or retrieving:

Weekday? [dropdown list] Weekend day? [dropdown list]

Obedience training:

Weekday? [dropdown list] Weekend day? [dropdown list]

Other playing activity (including dogs playing together):

Weekday? [dropdown list] Weekend day? [dropdown list]

[all dropdown list options are: None, 1-5 minutes, 5-15 minutes, 15-30 minutes, 30-60 minutes, 1-2 hours, Over 2 hours]

Is the quantity of exercise [dog name] receives each day:*
[dropdown list: Restricted due to your own time constraints, Restricted due to your own exercise ability, Restricted because of where you live, Restricted because [dog name] has a problem, As recommended by my dog breeder / my own experience, Unrestricted (you give as much exercise as you think your dog should have)]

Users need to click Save & Continue to proceed to next page

[After 10 month data entry, this section should be pre-populated with previous answers and a pop-up appearing (if they haven’t made any amendments) when users click to Save & Continue which reads: Thank you! You haven’t changed any of [dogname]'s exercise details. Do you want to continue to the next section or make changes to [dogname]'s exercise details? Select make changes/continue]

1.4 Pet Profile

* Required information

At present, how many times a day do you feed [dog name]?*
[help link reads: Don’t consider titbits, rewards or treats in this answer. “Throughout the day” means that food is available at all times]
[dropdown list: Once daily, Twice daily, Three times daily, More than three times daily, Throughout the day]

When do you feed [dog name]?*
[dropdown list: In the morning, In the evening, In the morning and evening, In the morning, lunchtime and evening, Throughout the day, Multiple times throughout the day]

What types of food do you give to [dog name]?*
[dropdown list: Dried food, Tinned food, A mixture of dried and tinned dog food, Home prepared food, Other]

[If Dried food selected]
Which dried foods do you feed [dog name]?*
[select one or multiple: Aldi, Asda, Bakers, Butchers, Brunos, Cesar, Chappie, High Life, Hills Iams, James Wellbeloved, Lidl, Pedigree, Royal Canin, Sainsburys, Tesco, Wagg, Wilsons, Winalot]
Not on the list?
[free-text box to enter product name]

How much dried food do you feed [dog name] in total each day? Enter a number, either in grams or ounces*
[help link to wording and pictures – see (3) below]
[free-text box to enter number, and dropdown list to select grams/ounces]

[If Tinned food selected, same as Dried food above except for wording as Tinned]

[If mixture of dried and tinned selected, have two sections as above, one for dried and one for tinned]

[If Home prepared, or Other, food selected]
What do you feed [dog name]?*
[free-text box to detail food]

How much home prepared food do you feed [dog name] in total each day? Enter a number, either in grams or ounces*
[help link to wording and pictures – see (3) below]
[free-text box to enter number, and dropdown list to select grams/ounces]

Does [dog name] also receive ‘titbits’? For example anything else your dog eats such as food off your plate, training treats, chews etc.?*
[select Yes/No]

What does [dog name] drink most days?*
[dropdown list: Water, Other. If ‘Other’ is selected, a free-text box appears to detail drink]

Users need to click Save & Continue to proceed to next page
